# Efficacy of Magnifying Endoscopy with Narrow-Band Imaging in the Diagnosis of Early Gastric Cancer and Gastric Intraepithelial Neoplasia

**DOI:** 10.5152/tjg.2024.23116

**Published:** 2024-04-01

**Authors:** Yanan Zhu, Kejian Wu, Fang Yu Wang

**Affiliations:** 1 Department of Gastroenterology, Jinling Hospital, Jinling Clinical Medical College of Nanjing Medical University, NanJing, China; 2 The Affiliated Hospital of Xuzhou Medical University, XuZhou, China

**Keywords:** NBI, magnifying endoscopy, gastric cancer, intraepithelial neoplasia endoscopy

## Abstract

**Background/Aims:**

Early diagnosis of gastric cancer can improve the prognosis of patients, especially for those with early gastric cancer (EGC), but only 15% of patients, or less, are diagnosed with EGC and precancerous lesions. Magnifying endoscopy with narrow-band imaging (ME-NBI) can improve diagnostic accuracy. We assess the efficacy of ME-NBI in diagnosing ECG and precancerous lesions, especially some characteristics under NBI + ME.

**Materials and Methods:**

This was a retrospective analysis of 131 patients with EGC or gastric intraepithelial neoplasia (IN) who had undergone endoscopic submucosal dissection and were pathologically diagnosed with EGC or IN according to 2019 WHO criteria for gastrointestinal tract tumors. We studied the characteristics of lesions under ME-NBI, compared the diagnostic efficacy of ME-NBI and white light endoscopy (WLI) plus biopsy, and investigated the effect of *Helicobacter pylori *infection on microvascular and microsurface pattern.

**Results:**

The diagnostic accuracy of ME-NBI for EGC, high-grade IN (HGIN), and low-grade IN (LGIN) was 76.06%, 77.96%, and 77.06%, respectively. The accuracy of WLI plus biopsy in diagnosing the above lesions was 69.7%, 57.5%, and 60.53%, respectively. The rate of gyrus-like tubular pattern was highest in LGIN (60.46%), whereas the highest rate of papillary pattern was 57.14% in HGIN and villous tubular pattern was 52% in EGC. Demarcation lines have better sensitivity for differentiating EGC from IN (92.06%).

**Conclusion:**

TheME-NBI has higher diagnostic accuracy for EGC than WLI plus biopsy. Demarcation lines and villous and papillary-like microsurface patterns are more specific as EGC and HGIN characteristics. The cerebral gyrus-like microsurface pattern is more specific for LGIN.

**Main Points**

- Magnifying endoscopy with narrow-band imaging (ME-NBI) has a higher diagnostic efficacy than white light endoscopy in the diagnosis of early gastric cancer and gastric precancerous lesions.
- Cerebral gyrus-like microsurface pattern was more specific for intraepithelial neoplasia, villous and papillary-like microsurface pattern was more frequently observed in early gastric cancer (EGC).
- Papillary and villous microsurface patterns were more frequently observed in *Helicobacter pylori* positive EGC and high-grade intraepithelial neoplasia patients, compared to negative ones.

## Introduction

The term early gastric cancer (EGC) indicates that carcinoma is confined to the mucosal or submucosal layer only, regardless of lymph node metastasis.^1^ The prognosis of gastric cancer is significantly related to the stage; the 5-year survival rate of patients with EGC can be greater than 90%. Gastric cancer has a complex pathogenesis and is the result of a combination of environmental, dietary, and genetic factors and *Helicobacter pylori* (HP) infection.^2^ The HP infection is an important factor for the development of gastric mucosal atrophy, intestinal metaplasia, and intraepithelial neoplasia.^3^ Correa’s cascade implies that gastric cancer develops in a multistep process from gastric mucosal atrophy, intestinal epithelial metaplasia, and dysplasia to progressive gastric cancer. According to the 2019 World Health Organization (WHO) Classification of Tumors of Digestive System (version 5), precancerous lesions are classified as low-grade intraepithelial neoplasia (LGIN) and high-grade intraepithelial neoplasia (HGIN). Detection of precancerous lesions greatly contributes to the recognition of EGC.

Magnifying endoscopy with narrow-band imaging (ME-NBI) is helpful for the diagnosis of EGC and precancerous lesions. Magnifying endoscopy with narrow-band imaging uses narrow-band imaging, involving narrow-band light, which is absorbed by intravascular hemoglobin, through a narrow-wave filter,^4,5^ clearly displaying the superficial microvascular (MV) morphology and microsurface (MS) pattern of the mucosa. With magnifying endoscopy (ME), magnifying lenses of different strengths are installed between the objective lens and a charge-coupled device that can magnify 60-170-fold. Muto et al^2^ proposed endoscopic diagnostic criteria for EGC, namely, a clear demarcation line (DL) plus an irregular MS pattern (IMSP) and/or an irregular MV pattern (IMVP).^6,7^ Several retrospective and prospective studies have confirmed the diagnostic value of ME-NBI in EGC;^6,8^ however, the diagnostic value of ME-NBI in intraepithelial neoplasia has been less thoroughly studied. In this study, we investigated the diagnostic efficiency of ME-NBI in EGC and intraepithelial neoplasia, compared the diagnostic accuracy of ME-NBI with white light imaging (WLI) plus biopsy in the diagnosis of EGC and intraepithelial neoplasia, and documented the characteristic manifestations of EGC and gastric intraepithelial neoplasia under ME-NBI and the effect of HP infection on EGC and intraepithelial neoplasia under ME-NBI.

## Materials and Methods

### Study Population

The study was conducted in the Gastrointestinal Endoscopy Center of the Affiliated Hospital of Xuzhou Medical University from March 2018 to March 2021. All enrolled patients had undergone endoscopic submucosal dissection (ESD) and were pathologically confirmed as having gastric intraepithelial neoplasia or differentiated EGC. There were 131 cases, comprising 63 EGCs, 25 HGIN cases, and 43 LGIN cases. The control group contained 41 patients diagnosed with chronic gastritis within the same time period. All patients in this group had undergone at least 1 ME-NBI examination and biopsy, with pathological examination showing chronic inflammation with or without intestinal metaplasia and atrophy.

Patients who could not tolerate the examination or were unwilling to cooperate with it were excluded. All participants gave written informed consent in accordance with the Declaration of Helsinki. The study was approved by the Ethics Committee of the Affiliated Hospital of Xuzhou Medical University (date: May 5, 2020; number: XYFY2020-KL045-01).

### Equipment and Reagents

The video endoscope used in this study was an Olympus GIF-H260Z/GIF-HQ290 and an electronic endoscope processing system, EVIS 290 Spectrum (Olympus, Tokyo, Japan). Simethicone, Pronase, and lidocaine hydrochloride mucilage were provided by Hubei Jumpcan Pharmaceutical Co. Ltd.

### Endoscopy Process

The endoscopist identified suspicious lesions under WLI endoscopy and biopsied them, as well as documented the height of the lesions and their surface characteristics (presence of nodules, granularity, surface tone, and luster).

#### Magnifying Endoscopy with Narrow-Band Imaging 

Patients underwent ME-NBI examination before ESD. The examinations were performed with propofol anesthesia. A soft black rubber cap was attached to the distal end of the endoscope so that the gastric mucosa was kept 2 mm away from the endoscope lens. During the examinations, mucus and foam were removed with a mixture containing 50 mL of water, 2 mL of Pronase, and 10 mL of simethicone oil.

Magnifying endoscopy with narrow-band imaging was set to the B8 mode to enable distant and near vision, and attention was given to changes in DL, IMVP, and IMSP. In particular, the DL was determined first. Images obtained during the examinations were stored.

**Figure 1.**
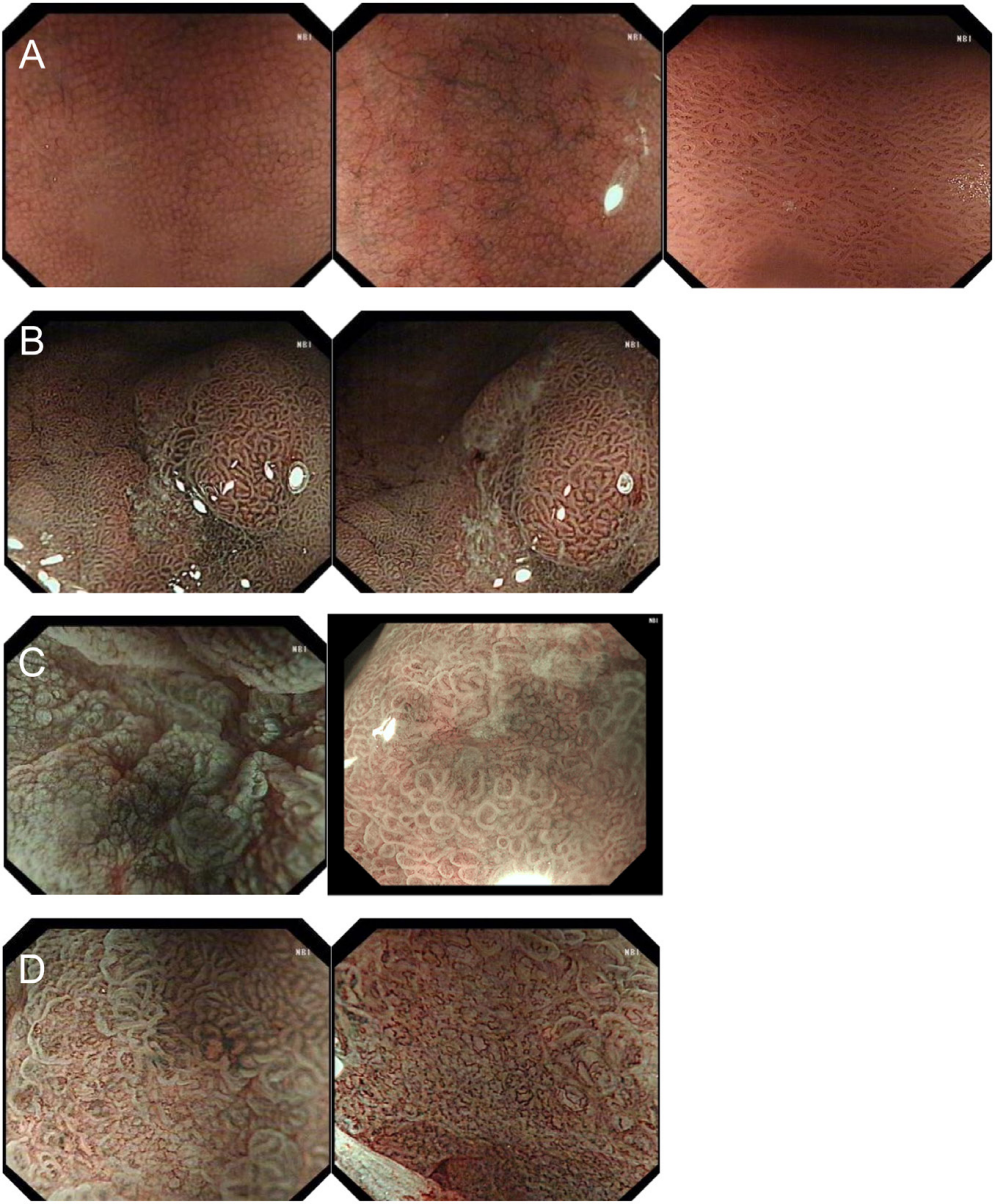
Features of gland duct in studied lesions. (A) Regular type fundic glands and dotted glandular ducts—the glandular structures are regularly arranged. (B) Cerebral gyrus-like ducts—the glandular structure is still circular but gradually dilated. (C) Papillary and villous glandular ducts—the gland ducts are arranged irregularly in size and shape. (D) Dilation and destruction of glandular ducts—the glands disappear or glandular structures are dilated in size.

The surface gland ducts were classified as follows, in accordance with Sakaki’s classification:^9^ 1, normal pattern (round pit); 2, cerebral gyrus-like pattern; 3, papillary, villous pattern; 4, destruction and dilatation of surface pattern; and 5, absence of microsurface pattern (Figure 1).

The operators had at least 5 years of endoscopic experience and at least 6 months of training in ME. The endoscopic images were also interpreted by 2 endoscopists with at least 10 years of endoscopic experience, both of whom were blinded to the clinical, histological, and serological findings. All 3 endoscopists aimed to identify the lesions’ DL and the morphology of the microvasculature and MS pattern. Conclusions were considered valid only with consensus between 2 or 3 of these endoscopists. These data were used to assess the sensitivity, specificity, accuracy, and positive and negative predictive values of the diagnosis of ME-NBI.

#### Pathological Analysis

Specimens of all lesions were obtained by ESD, and pathological diagnoses were made by 2 experienced pathologists who were blinded to the ME-NBI findings. The specimens were evaluated in accordance with the WHO Classification of Tumors of Digestive System (version 5) and Vienna Categories.^10^

#### Study Objectives

To retrospectively analyze the features of the MS pattern, MV pattern, and DL under ME-NBI for different types of lesions, compare the diagnostic accuracy of ME-NBI versus WLI plus biopsy, and investigate the effect of HP infection on EGC and intraepithelial neoplasia under ME-NBI.

### Statistical Analysis

The Statistical Package for the Social Sciences Statistics software 19.0 (SPSS Inc.; Chicago, IL, USA) was used for data processing and analysis. Categorical data were compared using the number of cases, and the chi-square test was used for comparisons between groups. The sensitivity, specificity, and positive and negative predictive values of the diagnostic results and accuracy were calculated using the 4-grid table method with 95% CIs. Two-by-two comparisons were performed using *t-*tests. Comparing post-ESD pathology with biopsy pathology, factors associated with upgrading were subjected to one-way analysis of variance.

## Results

### General and Clinicopathological Data of Patients with Early Gastric Cancer or Gastric Intraepithelial Neoplasia

The study cohort comprised 131 post-ESD patients, including 63 with EGC, 25 with HGIN, and 43 with LGIN. The control group consisted of 41 patients with chronic inflammation. The groups did not differ significantly in age, gender ratio (male to female, 1.69 : 1), or HP infection rate (42.45%). The locations of the lesions were the cardia (n = 43), gastric body/gastric angle (n = 57), and gastric antrum (n = 72). The morphology of the lesions was type 0-IIa in 64 cases, 0-IIb in 12 cases, 0-IIc in 49 cases, and mixed type in 47 cases. Regarding lesion size, 40 lesions were greater than 2 cm, and 91 were less than 2 cm. There were 54 cases of intramucosal carcinoma and 9 cases of submucosal carcinoma (Table 1).

### Comparison of the Diagnostic Accuracy of Magnifying Endoscopy with Narrow-Band Imaging and White Light Imaging Plus Biopsy in the Diagnosis of Early Gastric Cancer and Precancerous Lesions

There was no statistically significant difference between the specificity, positive predictive value, negative predictive value, or accuracy of ME-NBI and WLI plus biopsy for the diagnosis of LGIN. However, the accuracy, specificity, positive predictive value, and negative predictive value tended to be higher for ME-NBI than for WLI plus biopsy, indicating that the former may be more reliable.

There were no statistically significant differences in sensitivity, specificity, and accuracy of HGIN between NBI + ME and WLI plus biopsy, but in terms of numeral size, the specificity and accuracy were higher by ME-NBI than by WLI plus biopsy.

Magnifying endoscopy with narrow-band imaging had a significantly higher sensitivity, negative predictive value, and accuracy than WLI plus biopsy for the diagnosis of EGC (Table 2).

### Features of Early Gastric Cancer and Precancerous Lesions Under Magnifying Endoscopy with Narrow-Band Imaging

Demarcation lines were present in 92.6%, 68%, 48.84%, and 26.83% of cases in the EGC, HGIN, LGIN, and inflammation groups, respectively. The occurrence of DL among these groups was statistically significant.

The expression of IMVP differed significantly between the groups, with 72% in the HGIN group, 79.36% in the EGC group, and 34.89% in the LGIN group; the expression rate of IMVP was significantly different among these groups.

The difference in the occurrence of IMSPs was statistically significant among the LGIN, HGIN, and EGC groups. This finding implies that IMSP was useful for differentiating LGIN, HGIN, and EGC. Cerebral gyrus-like gland ducts were present more frequently in the LGIN group than in the other groups. The 2 MSs, papillary/villous surface pattern and gland duct dilation or disruption, were present significantly more frequently in the HGIN and EGC groups than in other groups, suggesting that these 2 MS characteristics play an important role in differentiating EGC and HGIN from other lesions (Tables 3 and 4).

### Effects of *Helicobacter pylori* Infection on Early Gastric Cancer and Gastric Intraepithelial Neoplasia

The rates of DL were essentially unaffected by HP infection. Papillary and villous tubular ducts were present more often in the EGC of the HP-positive group (62.92%) than in the negative group (52.78%). The destruction or dilatation of MSP was found in 33.33% and 27.78% of the EGC HP positive and negative groups, respectively, and the difference was not statistically significant. IMVP was found in 51.85% and 52.78% of EGC HP-infected and noninfected ones, respectively, and the difference was not significant. In HGIN and LGIN groups, papillary/villous glandular ducts were present significantly more often in HP-positive patients than in HP-negative patients (Table 5). Considering that HP infection may play a role in the process of heterogeneous proliferation of cancerous glandular ducts via unknown mechanisms, a chronic inflammatory response is probably produced through stimulation of cells and various cytokines.

### Single-Factor Analysis of Pathological Upgrading after Early Gastric Cancer Surgery

With post-ESD pathological diagnosis being the gold standard, we compared preoperative WLI plus biopsy and ME-NBI diagnoses with post-ESD pathology. Factors that resulted in upgrading included lesions larger than 2 cm, depressed type lesions, and lesions in the cardia (*P* < .05). However, lesion surface color and superficial elevation type were not significantly correlated with upgrading (*P* > .05). Early gastric cancer diagnosed by WLI plus biopsy were upgraded postoperatively in 32.06% of cases, whereas only 13.74% were upgraded in the ME-NBI group (Table 6).

## Discussion

Magnifying endoscopy with narrow-band imaging clearly displays the MP, microvasculature, and other structures, enabling assessment of the structural and vascular heterogeneity of lesions, which assists in determining the heterogeneity of their tissue and thus diagnosing what these lesions are.^11^ White light imaging is relatively poor for diagnosing EGC and intraepithelial neoplasia. In this study, one of our objectives was to compare the validity and reliability of ME-NBI and WLI plus biopsy in diagnosing EGC and gastric intraepithelial neoplasia.

We found that ME-NBI outperformed WLI plus biopsy in diagnostic efficacy. Factors associated with efficiency are as follows: taking biopsies from the correct site, lesion characteristics, and the ability of the gastroscopist to recognize microscopic lesions under endoscopy.^12^ In this study, ME-NBI was more accurate and had greater sensitivity and specificity for diagnosing EGC than WLI plus biopsy.

In the diagnosis of HGIN, the sensitivity and positive predictive values of ME-NBI and WLI + biopsy methods are both low, indicating that the diagnosis of HGIN is more difficult due to the unclear differences in cellular and structural abnormalities between HGIN and EGC. No statistically significant differences in sensitivity, specificity, or accuracy in diagnosing LGIN were found between the 2 methods. This is probably because of the insignificant glandular duct heterogeneity of LGIN under ME-NBI.

Papillary/villous glandular ducts were found to be 57.14%, 52%, and 25.58% in EGC, HGIN, and LGIN, respectively; glandular duct dilation/destruction was 30.16%, 36%, and 13.95% in EGC, HGIN, and LGIN, respectively. The above 2 morphological features of microsurface ducts were very valuable for diagnosing EGC and HGIN. The cerebral gyrus-like MP also had high specificity in differentiating LGIN from HGIN, and gyrus-like ducts were present in 60.46% of the LGIN group, which was significantly higher than that in the HGIN and EGC groups. The MP varies differently among these lesions due to the heterogeneity of glandular duct formation in differentiated carcinomas that grow and proliferate within the mucosa. These tubular ducts have irregular “skewed” shapes, and the degree of irregularity on the surface is positively related to the heterogeneity of the epithelial tumor formed by those ducts.^13^ In the same pathology group, IMVP is found significantly less frequently than IMSP, which indicates that alteration of the tubular ducts precedes and leads to extrusion degeneration of the microvasculature. Previous studies have also shown that ME-NBI is accurate in detecting precancerous lesions.^14-17^ In this study, IMSP was found more frequently in the HP-infected group than in the uninfected group for the same type of lesion, whereas the frequency of IMVP did not differ significantly between HP-infected and uninfected patients, implying that HP infection affects the morphology of mucosal epithelial microglandular ducts. Some studies have shown that HP infection leads to chronic inflammation and changes in various inflammatory factors, autoimmune reactions, and cytotoxins, leading to abnormal apoptosis or regenerative disorders, which eventually lead to heterogeneity of glandular ducts.^18,19^ The precise influence of HP infection on the formation of cancer epithelial glandular ducts needs further study.

We compared pathological diagnoses made on preoperative biopsies and postoperative pathology after ESD and performed a single-factor analysis. This showed that factors associated with postoperative pathological upgrading included a diameter larger than 2 cm, superficial depression type (0-IIc), and lesions located in the cardia (*P* < .05). Choi et al^20^ also found that the lesions being located in the cardia of the stomach are a risk factor for postoperative pathological escalation,which is consistent with our findings. The results of pathological examination of biopsies of lesions in the cardia may be affected by the limitations on sampling in this area imposed by inadequate endoscopic exposure. Relatively few studies have investigated whether HP infection is associated with pathological escalation. In the present study, we found no significant difference in the incidence of HP infection between the upgraded and nonupgraded groups, supporting the conclusion that HP infection is not significantly associated with pathologic upgrading. One study reported a 45.2% upgrading rate after ESD in patients with preoperative biopsy diagnoses of LGIN.^21^ In this study, 16 patients (32.06%) with biopsy diagnoses of LGIN were pathologically upgraded to HGIN or EGC after ESD, indicating the limitations of biopsies, the high possibility of missed diagnosis, and the need for close follow-up or diagnostic ESD for all LGIN lesions. The rate of pathological upgrading after ME-NBI was slightly lower than that after WLI plus biopsy, indicating the higher diagnostic accuracy of ME-NBI. Some studies have shown that unnecessary biopsies can be avoided when a lesion is not suspected to be cancer on ME-NBI.^22^ Despite the great potential of the clinical application of ME-NBI in the diagnosis of gastric mucosal lesions, there are still some limitations that need to be overcome.^23^ How to use ME-NBI to determine the depth of infiltration in differentiated EGC and the usefulness of ME-NBI in undifferentiated cancer are currently being studied. We recommend ME-NBI be used as a further examination option that can assist in determining the nature and borders of lesions after detection of suspicious superficial gastric lesions with white light endoscopy screening.

There are some limitations to our study. To investigate the effect of HP infection on the manifestation of lesions with ECG or HGIN under ME-NBI, we detected HP infection by only the 13C or 14C test and did not combine the infection with HP antibody, which may affect the accuracy of the results. The ME-NBI manifestation of the lesion is a morphological change, while the pathological result is a microscopic change, which is controlled by different doctors. Subjective factors, such as the experience of the observers, can affect the accuracy of the results. Our study is a single-center controlled study with a limited sample size and is influenced by researcher recall bias. The above factors will affect the accuracy and feasibility of the study, and future multicenter, large sample studies are needed.

## Figures and Tables

**Table 1.** Clinicopathological Data of Enrolled Patients

	EGC (n = 63)	HGIN (n = 25)	LGIN (n = 43)	Chronic gastritis (n = 41)	Total
Age	65.56 ± 10.48	65.08 ± 8.96	57.56 ± 9.24	58.45 ± 8.54	
Gender					
Male	41	14	25	28	108
Female	22	11	18	13	64
HP					
Negative	36	13	26	24	99
Positive	27	12	17	17	73
Site					
Cardia	17	10	10	6	43
Gastric angle + gastric body	18	5	9	25	57
Gastric sinus	28	10	24	10	72
General shape					
0-IIa	17	8	19	20	64
0-IIc	23	7	11	8	49
0-IIb	3	2	4	3	12
Mixed	20	8	9	10	47
Size					
<1 cm	13	10	27	-	50
1-2 cm	22	9	8	-	39
>2 cm	28	6	6	-	40
Infiltration depth					
Intramucosal carcinoma	54				54
Submucosal carcinoma	9				9

EGC, early gastric cancer; HGIN, high-grade intraepithelial neoplasia; HP, *Helicobacter pylori*; LGIN, low-grade intraepithelial neoplasia.

**Table 2.** Diagnostic Efficacy of Magnifying Endoscopy with Narrow-Band Imaging and White Light Imaging + Biopsy for Early Gastric Cancer and Precancerous Lesions

	Methods	Sensitivity (%)	Specificity (%)	PPV (%)	NPV (%)	Accuracy
LGIN	WLI + biopsy	67.44	71.43	64.45	74.07	69.70
	ME-NBI	60.47	88.00	74.29	79.52	77.96
HGIN	WLI + biopsy	48.00	58.90	25.00	82.35	57.50
	ME-NBI	52.00	86.02	48.00	86.02	77.06
EGC	WLI + biopsy	42.86	82.35	75.00	58.84	60.53
	ME-NBI	85.72	71.70	76.06	80.85	76.06

EGC, early gastric cancer; HGIN, high-grade intraepithelial neoplasia; LGIN, low-grade intraepithelial neoplasia; ME-NBI, magnifying endoscopy with narrow-band imaging; NPV, negative protective value; PPV, positive protective value; WLI, white light imaging.

**Table 3.** Features of studied lesions under ME-NBI

Pathology	Number of cases	Demarcation Line, n (%)	IMSP, n (%)	IMVP, n (%)
Chronic gastritis	41	11 (26.83)	3 (7.32)	4 (9.75)
LGIN	43	21 (48.84)	17 (39.53)	15 (34.89)
HGIN	25	17 (68.00)	22 (88.00)	18 (72.00)
EGC	63	58 (92.06)	59 (93.65)	50 (79.36)

*P* < .05 for demarcation line, IMSP, and IMVP.

EGC, early gastric cancer; HGIN, high-grade intraepithelial neoplasia; IMSP, irregular microsurface pattern; IMVP, irregular microvascular pattern; LGIN, low-grade intraepithelial neoplasia.

**Table 4.** Microvascular and Microsurface Features of Early Gastric Cancer, Precancerous Lesions, and Chronic Gastritis

	Degree of Lesion			
MS	EGC, n (%)	HGIN, n (%)	LGIN, n (%)	Inflammation, n (%)
Normal pattern	0 (0)	0 (0)	0 (0)	22 (53.66)
Cerebral gyrus like	4 (6.35)	3 (12.00)	26 (60.46)	16 (39.02)
Papillary/villous	36 (57.14)	13 (52.00)	11 (25.58)	3 (7.31)
Dilation/destruction	19 (30.16)	9 (36.00)	6 (13.95)	0 (0)
Disappearance	4 (6.35)	0 (0%)	0 (0%)	0 (0)
Irregular MV	33 (52.38)	13 (65.00)	12 (27.91)	4 (9.76)

EGC, early gastric cancer; HGIN, high-grade intraepithelial neoplasia; LGIN, low-grade intraepithelial neoplasia; MS, microsurface pattern; MV, microvascular pattern.

**Table 5.** Effect of *Helicobacter pylori* on Microsurface and Microvascular Features of Lesions

Effect of Helicobacter pylori on Microsurface and Microvascular Pattern of Leisons								
		DL, n (%)	Papillary/Villous, n (%)	Destruction/Dilation, n (%)	MS Disappearance, n (%)	IMVP, n (%)	FNP, n (%)	MV Blur, n (%)
EGC	HP (+)	26 (96.30)	17 (62.96)	9 (33.33)	3 (11.11)	14 (51.85)	7 (25.93)	6 (22.22)
	HP (−)	32 (88.89)	19 (52.78)	10 (27.78)	1 (2.78)	19 (52.78)	10 (27.78)	7 (19.44)
HGIN	HP (+)	8 (66.67)	8 (66.67)	3 (25.00)	0 (0)	7 (58.33)	3 (25.00)	2 (16.67)
	HP (−)	9 (69.23)	5 (38.46)	6 (46.15)	0 (0)	6 (46.15)	2 (15.38)	0 (0)
LGIN	HP (+)	9 (52.94)	8 (47.06)	2 (11.76)	0 (0)	5 (29.41)	2 (11.76)	0 (0)
	HP (−)	12 (46.15)	3 (11.54)	4 (15.38)	0 (0)	7 (26.92)	1 (3.84)	0 (0

DL, demarcation line; EGC, early gastric cancer; HGIN, high-grade intraepithelial neoplasia; HP, *Helicobacter pylori; *IMSP, irregular microsurface pattern; IMVP, irregular microvascular pattern; LGIN, low-grade intraepithelial neoplasia; MS, microsurface pattern; MV, microvascular pattern.

**Table 6.** Single-Factor Analysis of Factors Associated with Postoperative Pathological Upgrading

	Pathological Upgrading Group (n = 36)	Pathological Nonupgrading Group (n = 27)	*P*
Age	66.14 ± 9.65	62.25 ± 7.78	
Gender, n (%)			
Male	22 (61.11)	19 (70.37)	
HP, n (%)			
Positive	15 (41.67)	12 (44.44)	
Location, n (%)			
Cardia	11 (30.56)	6 (22.22)	<.05
Gastric angle + gastric body	9 (25.00)	9 (33.33)	
Gastric sinus	16 (44.44)	12 (44.45)	
Gross morphology, n (%)			
0-IIa	9 (25.00)	9 (33.33)	
0-IIc	15 (41.67)	8 (29.63)	<.05
0-IIb	1 (2.78)	2 (7.41)	
Mixed	12 (33.33)	8 (29.63)	
Mucous membrane color, n (%)			
Redness	34 (94.44)	24 (88.89)	
Lesion size, n (%)			
<2 cm	17 (47.22)	18 (66.67)	
>2 cm	19 (52.78)	9 (33.33)	<.05
Postoperative upgrade rates (%)			
ME-NBI	13.74		
WLI	32.06		

HP, *Helicobacter pylori; *ME-NBI, magnifying endoscopy with narrow-band imaging; WLI, white light imaging.
